# Compassionate Discourses: A Qualitative Study Exploring How Compassion Can Transform Healthcare for 2SLGBTQ+ People

**DOI:** 10.1177/10497323221110701

**Published:** 2022-06-23

**Authors:** Phillip Joy, Andrew Thomas, Megan Aston

**Affiliations:** 1 Department of Applied Human Nutrition, 3684 Mount Saint Vincent University, Halifax, NS, Canada; 2 School of Nursing, 3688 Dalhousie University, Halifax, NS, Canada

**Keywords:** compassion, queer, 2SLGBTQ+, poststructuralism, qualitative, discourse analysis

## Abstract

Compassion can be seen as a necessary, but often lacking, concept and practice in healthcare. Due to the cis-heteronormative nature of societies, people who identify as Two-Spirit, lesbian, gay, bisexual, transgender, queer (2SLGBTQ+) often experience health disparities and disparities in accessing compassionate healthcare. We aimed to explore the meanings of compassion in healthcare for Canadian 2SLGBTQ+ people. Using a poststructuralist framework, 20 self-identifying 2SLGBTQ+ participants were interviewed. Data was analyzed through discourse analysis. Three main discursive considerations are discussed, including (1) meanings and expectations of compassion in healthcare, (2) compassionate healthcare is not guaranteed, and (3) prescription for care: self-compassion for healing and health. The results provide insights into how compassionate healthcare is framed for 2SLGBTQ+ participants and how compassion is often lacking for them due to discourses of cis-heteronormativity and healthism.

Compassion is a concept that has been defined in many ways by philosophers, theorists, and social scientists. Strauss et al. (2016) reviewed several definitions of compassion:

“The feeling that arises in witnessing another's suffering and that motivates a subsequent desire to help” (Goetz et al., 2010, p. 351).

“Being touched by the suffering of others, opening one's awareness to others' pain and not avoiding or disconnecting from it, so that feelings of kindness towards others and the desire to alleviate their suffering emerge. It also involves offering non-judgmental understanding to those who fail or do wrong” (Neff, 2003a, p. 86–87).

“An orientation of mind that recognises pain and the universality of pain in human experience and the capacity to meet that pain with kindness, empathy, equanimity and patience” (Feldman & Kuyken, 2011, p. 145).

These meanings position compassion as recognizing the universality of suffering and pain of human experiences while also positioning compassion as an act of non-judgmental practices to alleviate suffering. Through these definitions, we can understand compassion as an important component of quality healthcare. Within healthcare compassion has been noted to be a priority (Sinclair et al., 2016). Qualities of compassionate healthcare professionals has been noted to include honesty, openness, genuineness, and a desire to help others that lead to actions that help patients. The abilities to listen, to be accepting, and the capacity to get to know and understand a person have also been described as qualities of compassionate healthcare professionals (Kneafsey et al., 2016; Sinclair et al., 2016).

Compassion within healthcare can take many forms. First, healthcare professionals may use compassion in their practice through acts of kindness and respect. Compassion in healthcare has been shown to have positive effects on patients’ health (Sinclair et al., 2016) and compassionate communication may help patients’ emotional health and symptom resolution (Durkin et al., 2018; Roter et al., 1998; Stewart, 1995). As little as 40 seconds of compassionate healthcare practices, including acknowledging concerns, expressing partnership and support, and validating emotions, have been shown to make differences in patients’ experiences and health (Fogarty et al., 1999).

Compassion in healthcare may also be present at the organization level. Organizational compassion refers to the implementation of ethical measures and policies to enhance compassion within the organization and by the individuals within it (Fotaki, 2015). In a study with healthcare professionals, it was found that healthcare systems and healthcare organizations have important roles to play in fostering the use of compassion by healthcare professionals through learning and training opportunities and by making compassion a core competency and priority for their healthcare professionals (Sinclair et al., 2020). Improving institutional healthcare policies and practices to be more compassionate can also improve patient outcomes (Simpson et al., 2020).

A third form of compassion within healthcare is compassion practiced by the patients and directed towards themselves. Neff (2003b) described self-compassion as encompassing three elements: self-kindness or being less harsh in judging oneself, mindfulness or balancing painful thoughts and feelings with an awareness of not over-identifying with those painful thoughts and feelings, and common humanity or seeing one’s experiences as shared experiences with others rather than isolating or separating experiences. Self-compassion has been shown to be a powerful tool in dealing with mental health issues, anxiety, depression, eating behaviors, and treating addictions (Braun et al., 2016; Ferrari et al., 2019; Phelps et al., 2018). Self-compassion has also been suggested to create a “proactive attitude towards self-care” (Torrijos-Zarcero et al., 2021, p. 930) and an increase in health-promoting behaviors (Misurya et al., 2020).

Accessing and receiving compassionate healthcare, however, is often not possible for many groups, including Two-Spirit, lesbian, gay, bisexual, transgender, queer, and other sexual identities, such as pansexual or asexual (2SLGBTQ+) individuals. Cis-heteronormativity, or the assumption that all people are straight and either distinctly man or woman, create many health issues for 2SLGBTQ+ people, often at disproportionate rates compared to the wider population (Casey, 2019). The Society at a Glance Report found that only half of the Canadian population are accepting of 2SLGBTQ+ people and more than one out of three 2SLGBTQ+ people felt personally discriminated against at some point (OECD, 2019). Such social values contribute to many of the health disaparities within 2SLGBTQ+ communities, including mood and anxiety disorders (Baams et al., 2021; Mueller, 2020; Pakula et al., 2016), homelessness (City of Toronto, 2013), body image and eating disorders (Parker & Harriger, 2020), suicidality (Brennan et al., 2010; Scanlon et al., 2010), and HIV (Haddad et al., 2019).

Cis-heteronormativity is also a systemic issue within healthcare systems (Fish & Bewley, 2010) and influences the way 2SLGBTQ+ people access and receive healthcare. McGlynn et al. (2020) report that cis-heteronormative assumptions are a major barrier to healthcare. Often sexual orientation is ignored because it is not considered a relevant topic to healthcare or viewed as a taboo not to be discussed (Fish & Bewley, 2010; McGlynn et al., 2020). This may lead many to experience fear about disclosing sexuality and gender identity, partners, and sexual practices, as well as contribute to feelings of not being known. For example, queer, lesbian, and bisexual women continue to experience homophobia and invisibility in the health care system in Eastern Canada (Fredericks et al., 2017). Older adults in Ireland reported having a negative reaction from their healthcare provider after disclosing sexuality (McCann & Sharek, 2014). The fear of being denied safe and quality healthcare on account of being 2SLGBTQ+ still persists for many 2SLGBTQ+ individuals who are then left to avoid doctors who do not make them feel comfortable and to avoid uncomfortable subjects or disclosures (Harbin et al., 2012).

Additionally, the social discourses of cis-heteronormativity have created attitudes, values, and beliefs for many healthcare professionals that are opposed to compassionate care. Fear, ignorance, prejudice, and the judgmentalness of nurses have been noted to play a role in the care of 2SLGBTQ+ people, particularly with those living with HIV/AIDS (Surlis & Hyde, 2001; Vaughan et al., 2020). A Canadian report indicates that as many as one in five sexually diverse men have experienced some form of sexual orientation, gender identity, or gender expression change efforts and that approximately 40% of these men have experienced conversion therapy, an abusive psychological intervention (Sex Now Survey results reveal prevalence of change efforts, 2020). The continued banning of blood donations in Canada from men who have sex with men is discriminatory and reflects persistent pathological views of gay men and other men who have sex with men (Grace et al., 2019). Currently, Gender Disorders and Paraphilias are still listed as disorders in the DSM 5. This pathologizes diverse expressions of sexuality and gender, and further stigmatizes these individuals (Daley & Mulé, 2014). Such issues are often compounded by a lack of training (Bell et al., 2010; Carabez et al., 2015; Obedin-Maliver et al., 2011). Physicians have reported a lack of advanced knowledge of 2SLGBTQ+ issues and that more training would be well received (Gentile et al., 2021). In Canada, there has been recognition for the need of more 2SLGBTQ+ health recommendations, training modules for health professionals, 2SLGBTQ+ health data collection, and more funding for 2SLGBTQ+ health organizations and research (Casey, 2019).

With this research, we aim to contribute to the growing body of compassionate healthcare literature by exploring the various meanings of compassion in healthcare for 2SLGBTQ+ individuals. Specifically, we seek to more fully understand how the beliefs, values, and experiences of compassion in healthcare are constructed through social discourses of cis-heteronormativity and healthism for 2SLGBTQ+ people.

## Methods

### Theoretical Perspectives

This research is rooted in poststructuralism, specifically the works of Foucault (1977, 1978) that explores how the experiences, identities, and lives of people are socially constructed through relationships of discourse, knowledge, and power. Discourse can be thought of as interconnected systems of social meanings and practices “that systematically form the objects of which they speak” (Foucault, 1972, p. 49). Within society, there are many different discourses that are constantly merging, overlapping, and being re-created as people collectively think about and speak about the world in which they live (Fendler, 2010). Discourses are not simply what is spoken within society but also that which is not spoken. Discourses are how we know things and, ultimately, how our experiences, beliefs, values, and practices are shaped (Foucault, 1977, 1978). Some discourses become dominant discourse within society, while other discourses become repressed, unheard, or marginalized. According to Foucault (1977, 1978), it is the relations of power within society that influence and shape social discourses, and ultimately the experiences of people. The meanings of compassion ascribed by people are therefore constructed through social discourses. The experiences of compassion and how people practice compassion within healthcare are also socially constructed through discourses. We, therefore, believe the lens of poststructuralism is an appropriate theoretical lens to explore the various meanings of compassion in healthcare for 2SLGBTQ+ individuals.

### Recruitment and Data Collection

Participants were recruited through ads placed on various social media platforms (Facebook and twitter), as well as ads to 2SLGBTQ+ community organizations and compassion networks (i.e., The Charter of Compassion) within Canada. To be eligible, participants were required to be 19 years of age or older, reside in Canada, self-identify as 2SLGBTQ+, and be interested in discussing their beliefs, values, and experiences of compassion.

Twenty potential participants were sent an informed consent that contained the aims of the research, risks and benefits of participating, and confidentiality protocols. Once the potential participants signed and returned the informed consent they were scheduled for an interview. Interview questions were framed through a post-structural theoretical lens and guided by the insider knowledge of the 2SLGBTQ+ researchers (Hayfield & Huxley, 2015; LaSala et al., 2008). Open-ended interview questions were designed to allow participants to reflect and discuss their belief, values, and practices relating to compassion. Demographic questions relating to gender, sexual orientation, race, and age were added as part of the interview guide. The demographic questions were also open-ended to allow participants to self-identify.

Participants took part in virtual interviews that lasted approximately 60–90 min using Teams™ (Microsoft, version 1.4.00.19572, Washington, 2017). All online semi-structured interviews were done by the same research team member. Interview guides were not provided prior to participating. All interviews were recorded using Microsoft Teams with notes taken during the interviews by the interviewer to supplement the recording. Interview recordings were transcribed by the interviewer and anonymized for analysis. Participants reviewed and approved their transcripts. Data collection took place from May through September 2021.

### Ethical Considerations

Researchers who work with groups who have been historically marginalized, such as 2SLGBTQ+ groups, need to ensure ethical protocols to research are just, fair, and protect the welfare of their participants. The risks of this research were considered minimal. In our ethics review, we identified the following as potential risks: (1) Psychological or emotional discomfort (e.g., anxiety, stress, loss of confidence, regret for disclosing personal information), (2) Social repercussions (e.g., possibility of marginalization, being negatively judged by peers or employer), and (3) Economic inconveniences (e.g., expenses incurred for participation, loss of income during time of participation). To address the risks of psychological or emotional discomfort a list of community service organizations was provided to participants as part of the informed consent process. Participants were advised to contact their own health care professional or the community service organizations if they needed to discuss their experiences. To address the risk of social repercussions, all personal data was removed during the transcription process and all participants were given an ID number. Finally, to minimize the economic inconveniences, participants were given an honorarium ($50 CAN) for participating in the study. This research was cleared by the research ethics board.

### Data Analysis

Interviews were analyzed using Foucauldian discourse analysis. As Arribas-Ayllon and Walkerdine (2008, p. 110) warned “there are no set rules or procedures for conducting Foucauldian-inspired analysis.” However Foucauldian discourse analysis involves systematic and critical examinations of data that look beyond the surface meaning to situate the texts within their historical, political, cultural, and social contexts, paying particular attention to the way language and discourses shape the values, beliefs, and experiences of participants (Foucault, 1977, 1978).

For this research, the process involved the researchers independently reviewing the interview transcripts multiple times. Each researcher coded the beliefs, values, and practices of the participants in relation to compassion. After the individual review process, researchers collaboratively discussed the data. Conflicts and similarities about codes were discussed between members of the team and resulted in a return to the interview data for another round of independent review and subsequent round of collaborative discussion. During this round of discussion, the codes were also reviewed in the context of relevant social discourses (refer to Findings section), drawing connections between these discourses and the words of the participants to gain further insight into the meanings of compassion. This discussion resulted in the construction of discursive considerations. We present three discursive considerations in the Findings section. All analysis was done in Microsoft Word and Microsoft Excel.

### Research Evaluation

Richardson and St Pierre (2005) envisioned evaluation for research was framed within the poststructural paradigms not as a triangle that is typical for much qualitative research, but rather as a crystal, stating that

the central imaginary for ‘validation’ … is not the triangle – a rigid, fixed, two-dimensional object. Rather the central imagery is the crystal, which combines symmetry and substance with an infinite variety of shapes, substances, transmutation, multidimensionalities, and angles of approach… crystals are prisms that reflect externalities and refract within themselves…what we see depends on our angle of response’ (p. 963).

Our angles of approach within this research included the following: (a) data included the interview transcripts and interviewer notes, (b) data were independently coded by each team member, (c) discursive considerations were finalized through a collaborative process, (d) researchers asked interview participants to check their data for meaning, and (e) we provide a reflection on positionality as researchers.

We acknowledge and present our perspectives and our identities that frame this research. Two authors are faculty members within health disciplines, one in dietetics and the other in nursing. Two authors identify as members of the 2SLGBTQ+ community and the third author identifies as cis-gendered, heterosexual, women and has worked with the 2SLGBTQ+ communities. All authors are committed to creating more discussions and knowledge regarding the experiences of 2SLGBTQ+ folks who face inequalities within healthcare systems and are dedicated to improving the health and wellbeing for 2SLGBTQ+ individuals through their work.

## Findings

### Participants and the Framing of Social Discourse

In total, 20 2SLGBTQ+ self-identifying individuals were recruited and partook in the interviews. The participants’ demographic details are found in Table 1. We present three main discursive considerations that are framed by two social discourses. The first social discourse (Discursive Considerations 1 and 2) was cis-heteronormativity, or that assumptions that all people are straight and identify as distinctly a man or a woman, contribute to many health disparities for 2SLGBTQ+ people and is also a systemic issue within healthcare systems that influences the way 2SLGBTQ+ people access and receive healthcare (Fish & Bewley, 2010), often in ways opposed to compassionate care. The second social discourse was healthism (Discursive Consideration 3). Healthism suggests that the individual, rather than society, is solely responsible for health (Crawford, 1980; McGregor, 2001; Thorsen, 2010). In the healthism discourse, good health becomes based on individual morality, determination, hard work, good citizenship, and the ability to follow health recommendations instead of being a reflection of political, social, and cultural contexts such as cis-heteronormativity, racism, and sexism (McGregor, 2001; Thorsen, 2010). The assumptions of healthism dictate that people focus on self-interest rather than mutual interests and shared humanity. Healthism seems to oppose most definitions of compassion and compassionate healthcare.

**Table 1.** Participant Demographics.

ID	Age	Province	Self-identified ethnicity	Self-identified sexuality	Self-identified gender
1	24	Nova Scotia	White	Queer	Fem-leaning nonbinary
2	31	Nova Scotia	White	Queer	Cis woman
3	48	Nova Scotia	White	Lesbian	Nonbinary/Agender
4	50	New Brunswick	White	Gay	Genderfluid
5	24	Nova Scotia	White	Bisexual	Cis woman
6	32	British Columbia	White	Lesbian	Cis woman
7	21	Nova Scotia	White	Queer	Trans man
8	39	Nova Scotia	White	Queer	Cis woman
9	26	Ontario	White	Queer	Trans woman
10	22	Nova Scotia	White	Bisexual	Cis man
11	36	British Columbia	Latino	Gay	Cis man
12	53	Ontario	White	Lesbian	Cis woman
13	42	Nova Scotia	White	Gay	Cis man
14	22	Alberta	Asian/white	Bisexual/Asexual	Genderfluid
15	26	British Columbia	Multiracial	Queer	Queer
16	54	British Columbia	White	Queer	Nonbinary
17	34	Ontario	Mixed	Gay	Cis man
18	—	Ontario	Black	Bisexual	Cis woman
19	25	Ontario	Black	Bisexual	Cis woman
20	44	Ontario	Mixed	Queer	Cis woman

### Discursive Consideration 1: Meanings and Expectations of Compassion in Healthcare

All participants discussed the meanings of compassion for themselves and for members of 2SLGBTQ+ groups. However, some participants found the concept of compassion was difficult to define. For example, Participant 4 said “I didn't know how to put that into words” and Participant 7 noted that compassion was “such a loose concept… I know what that is. I can point it out when I see it… but I don't know how to describe it.” As this participant exemplified, although the meanings of compassion were hard to verbalize compassion is something that is recognizable, something that can be pointed out and shown to others. Most participants also believed compassion was something that was integral to 2SLGBTQ+ healthcare. As Participant 7 noted, “good healthcare has to have compassion at the core.”

Although compassion itself was difficult to describe in words, many participants, however, did have beliefs about what compassion looked like in healthcare. Comfort was the first expectation of compassionate healthcare.

I'm more comfortable going to queer healthcare practitioners than I would be straight ones because I feel automatically more comfortable and I think making your patient feel comfortable and feel like they're listened to is a huge part of compassion in healthcare, and I think that that is hugely helped by seeing people like yourself reflected back at you in healthcare (Participant 7).

This participant demonstrated how they had experienced a dichotomy between straight and queer healthcare practitioners and used their agency to choose to seek care from a healthcare practitioner who made them feel comfort. Comfort was viewed as knowing they would be listened to and in seeing themselves reflected in their practitioner.

In addition to comfort, safety was also believed by many participants to be a part of compassion. As participant 3 said compassion is “important for quality of life and for safety.” This participant further provided an example of how cis-heteronormative discourses can lead to people not feeling safe.

Being anti-trans isn't a difference of opinion it can lead to people being unsafe and I think if there was more compassion and understanding… maybe we wouldn't have the kind of neutral people saying it's ‘OK, it's just a difference of opinion.

For this participant compassion was a way to move beyond cis-heteronormative discourses and a means to build more understanding about trans experiences in healthcare.

Another participant, who worked in the field of Indigenous health, also discussed the relationship between compassion and safety.

There are those who are allies, and there are those who are really doing everything that they can to unlearn, relearn, support, to be open, to be compassionate, but again also doing a lot of work in Indigenous engagement and health we're constantly pushing for cultural safety training and emphasizing the importance of cultural safety training and bringing into the spaces that we work and occupy so that people have understanding of context and understanding of how certain health concerns might arise. But I bring that up because I think it is parallel to 2SLGBTQ+ health (Participant 20).

This participant highlights the importance of cultural safety as part of the expectation for compassionate 2SLGBTQ+ healthcare. Providing healthcare professionals with training and opportunities to unlearn knowledge construction through (western) cis-heteronormative and healthism discourses was integral for compassionate healthcare systems and practitioners. However, as Participant 20 further noted, fostering both compassion and cultural safety in healthcare is challenging, complex, and not easy.

It's challenging. I don't really have an answer. I think that we can put the human face in place, we can put the stories in place, we can put the trainings in place, we can open spaces for dialogue, we can have pride and national Indigenous People’s Day, we can do all these things, but people have to have a willingness to change. People have to be willing and open to learning, and I think the challenge is creating a space that people feel like they can safely express their biases and then kind of work through that (Participant 20).

This participant believed it was important to create spaces for health professionals to explore, express, and work through their biases produced by (western) cis-heteronormative and healthism discourses that create unsafe spaces within healthcare systems. Challenging these discourses were viewed as integral to shifting unsafe and uncompassionate healthcare systems to ones that are safe and compassionate.

Several participants also described the use of inclusive language as part of compassion. Participant 1 described how “non-judgmental language and being really careful around that” was important for compassionate healthcare. As Participant 1 further explained, if someone uses language that is “a little bit judgy or shows they’re not quite grasping where you’re at, I think that can be really, really jarring and can make it hard to want to ask for compassion from that person again.” Participant 1 noted that language that is judgmental and language that indicates a healthcare professional does not understand them as a 2SLGBTQ+ person can be harmful and can often result in barriers for many seeking compassionate healthcare. Awareness of gendered language and pronouns was also believed by many participants to be a part of compassionate healthcare. Participant 6 described how they believed, “…not having a gender-neutral pronoun option or something like that on a form… is a microaggression.” The participant believed such acts were microaggressions because such acts are rooted in cis-heteronormative construction of medical forms and healthcare systems that demonstrate a lack of empathy and understanding for 2SLGBTQ+ people. The participant further explained that such microaggressions are deeply present in the daily life of many 2SLGBTQ+ people and why understanding and compassion is needed in the language of healthcare.

Participants believed that social discourses of cis-heteronormativity created shared experiences and trauma for many 2SLGBTQ+ people. An awareness and an understanding of shared experiences was understood by many participants to be a component of compassion. As Participant 1 described there are “…many traumas that everyone who’s queer has to go through in our society” and that compassion is about “really accepting and caring about people for who they are, regardless of anything else.” Being compassionate was looking beyond the cis-heteronormative assumptions within society that “other” 2SLGBTQ+ people. Compassion was about accepting and caring for people regardless of sexual orientations and genders. Participant 15 also believed compassion was “having a certain amount of graciousness towards others and ourselves and people in our community because we’ve undergone a lot of different traumas.”

Participant 15 recognized that compassion was required to help people heal from the traumas from the “messy” socially constructed traumas.

Traumas were noted to take many different forms that often lead to many struggles for 2SLGBTQ+ people. Participant 16 shared their experiences of trauma.

When I was a kid I was abused at home but also at school I was bullied terribly because I'm nonbinary and when I was little the kids would call me “IT” and I was totally ostracized… I would just pretend that I was a boy, it was easier because everyone assumed I was a boy anyway… And the teachers. Yeah, I mean the teachers didn't call me “IT”, but they actually joined in on the bullying a couple times for sure like blatantly… it was such a cruel environment. It was just so normal then for just everybody to be mean.

Cis-heteronormative norms about gender created trauma for Participant 16 as they went through school. They were bullied by other students but also by their teachers, although it was less blatant by the teachers. They noted that It was a “cruel” environment for them to grow up in and left many emotional and mental wounds for them to struggle with throughout their life. As other participants confirmed trauma can often negatively influence people’s lives, health, and wellbeing. Participant 4 noted that “many of us have personal histories of childhood through our personal history, historical traumas, and then those traumas come out in our adult lives with our jobs or work or relationships or situations… and as we’re unpacking all of this stuff and feeling like we’re broken.” Trauma from cis-heteronormative discourses can lead many 2SLGBTQ+ people to feel broken, and in turn shaping their health. Several participants discussed very specific examples. For example, Participant 7 believed trauma was associated with “higher rates of addiction, there’s more homelessness, there is high rates of everything really: suicide and mental health issues” whereas Participant 15 made the connection between trauma, body image and eating disorders, saying, “makes sense to me that eating disorders and body image would really affect us… like as a trauma response.” This participant saw trauma for many 2SLGBTQ+ people was created by gender norms related to the development of body image concerns and eating disorders. They also noted that many doctors did not understand this connection and, by not understanding, were providing non-compassionate care.

Comfort, safety, inclusive language, and awareness and understanding of shared trauma were ways participants understood compassion and how they expected healthcare to be compassionate. Participant 2 provided an example of aspirational compassionate healthcare when they described their experiences during the COVID-19 pandemic. They believed that “COVID brought out this huge experience of shared humanity among all kinds of different people… a lot of compassion showed through in those first early months, where we’re all in this together.” They continued to describe one such experience of compassion provided by their doctor.

Going to a doctor's appointment and because of COVID having to go alone and not being able to bring a support person with me and then having the provider actually read my file and see that I have a partner and see their name and they asked me if I wanted to have them on FaceTime during it. To me that was very compassionate… It was so easy and such a simple thing to offer that made a huge difference for me… They recognize that during a health appointment it is very important for you to have someone... like they do see my partner as valid and important in my life and I don't have to fight for that in any way.

The act of moving beyond cis-heteronormative constructs of relationships and recognizing the partners of 2SLGBTQ+ people by healthcare professional was viewed to be an act of compassion by the participant. The participant believed the doctor understood them and recognized their need to have their partner present without any assumptions or questions about gender and sexual orientation. The participant found compassionate healthcare through comfort, safety, inclusive language, and awareness and understanding of shared trauma.

### Discursive Consideration 2: Compassionate Healthcare is Not Guaranteed

Many participants sought compassion from health professionals and believed that healthcare systems should be designed with compassion as a core principle. As Participant 2 explained, when “you’re accessing the healthcare system you would expect compassion from the healthcare system. In an ideal world and I know a lot of people, queer or not, don't have that experience.” As this participant continued, they noted that “compassion isn’t guaranteed in healthcare, but when it’s found its celebrated (Participant 2).” Participant 3 “hoped that the mental health profession would be [compassionate] but [they] find that it's largely the opposite.” Participant 5 noted that compassionate healthcare professionals can “hit or miss… it really depends on where you live and stuff like that too cause I feel like there isn’t a lot of help especially in rural areas… they [healthcare professionals] don’t have that kind of training [2SLGBTQ+ training]". Participant 19 believed that

there's a gap that exists in terms of sexual health of the queer community. I think it should be more specific, like there should be centers for the queer community only so that they are able to feel like they can open up to someone without judgment, someone who really understands.

Other participants also discussed how Canadian healthcare institutions do not provide the things that the participants believed make for compassionate healthcare.

I think that there is a lot of heteronormative assumptions… I've worked in what would be considered to be safe spaces, in hospitals and healthcare teams… I have a fear there won't be compassion there. I have a fear there will be judgment. I have a fear that whether that's true or not I actually don't know, but it shapes my experience (Participant 20).

This participant challenged the belief that the healthcare system is a compassionate space. Safety, as previously discussed, has particular meanings in healthcare for 2SLGBTQ+ people. Cis-heteronormative assumptions were viewed by this participant as opposite to safe, and by connection, compassionate healthcare. Cis-heteronormative discourses often create fear and worry for many 2SLGBTQ+ people. As this participant exemplifies, many 2SLGBTQ+ people worry they will not find compassion from health professionals and within the institutions of healthcare. Compassion that is necessary for healing.

A few participants used the HIV/AIDS crisis as an historic example of non-compassion in the healthcare system. Participant 12 described their experience in the early AIDS crisis.

When I was in my 20s, the AIDS crisis was at its peak, and although in the long run I think that inspired compassion among the general public at the time there was a lot of negativity. A lot of blaming of people, blaming of behaviors. A lot of religious nastiness. So, overtime that has changed and I think media had a lot to do with it and the organization of the queer community during the AIDS crisis, and I think more visibility, humanized people to the general public in a way that hadn't happened before.

Although this participant did not explicitly comment on healthcare professionals they did note the overall lack of compassion within society for people living with HIV/AIDS and how the 2SLGBTQ+ communities organized together to challenge negative ideas being perpetuated through cis-heteronormative discourses that were pathologizing to 2SLGBTQ+ people. Advocacy and challenging these social discourses were ways that changes were made. In other words, society and healthcare became more compassionate to people living with HIV/AIDS.

Participant 4 highlighted that although in Canada the AIDS crisis has changed dramatically over the years some of their friends that “lived through the hard-fought battles” of the early AIDS crisis “continue to have a lot of pain” and that progress towards more compassion, especially in healthcare, is still needed.

### Discursive Consideration 3: Prescription for Care: Self-Compassion for Healing and Health

All participants believed that self-compassion was important for the health of 2SLGBTQ+ people, in particular mental health. Despite the importance of self-compassion, many participants found it difficult to do.

I really have a hard time having any self-compassion for myself… I’m very very hard on myself. That's something I've been told by people time and time again is to have compassion for myself. So, I feel like it's important that people have that but I personally I struggle with it (Participant 17).” Difficulties in having self-compassion were often viewed by participants to be interconnected with the traumas people often experience as members of 2SLGBTQ+ groups.

Participants spoke about self-compassion in relation to healing from personal traumas created by societal cis-heteronormative discourses. Participant 11 provided insight into this belief.

How can you have self-compassion when you have an open wound your whole life? OK, you can treat it, you can get the band aid. You can get some medication, try to take care of it, and try to pamper it a little bit. You know try to be good to yourself and sadly that cut or that wound becomes poisonous, becomes pussy, becomes infected, becomes rotten, becomes bad. I have experience that. I have seen that in our queer community.

The open wounds referred to by this participant are the wounds that 2SLGBTQ+ people may suffer due to stigma and discrimination. Interestingly, many participants found it easier to be compassionate with other 2SLGBTQ+ people than to themselves. Participants described having a hard time showing the same level of compassion for themselves. Participant 7 noted that

maybe it's just contending with society as a whole not being quite as positive. You can almost push that aside and overlook it and be like ‘I'm with you 100%’ for other people, but I feel maybe your doubts might be a little bit more there for yourself… like a kind of internalized homophobia, transphobia.” For this participant, and many others, it was easier to recognize another’s hurt and suffering and to be compassionate towards them. Internalized homophobia and internalized transphobia (terms that we further explore in the discussion) were believed to be part of the reason why self-compassion is difficult.

Several participants described experiences with healthcare professionals who prescribed self-compassion practices for improving their health and feelings of wellbeing. One participant recounted their experience with a therapist who relayed the importance of self-compassion to them.

‘I will be your therapist… I need for you to understand the importance of compassion and the importance of self-compassion… We can't control what others are doing to us, but we can definitely control what it is that you're doing to you.’ So, I had to learn a whole new therapeutic language [the language of self-compassion] (Participant 5.)

Another participant described a similar instance with a health professional. “He was like ‘you know what it sounds like? You need to learn to love yourself’ and I was like I don't know how that was even possible” (Participant 16). Another participant discussed going to therapy and the use of affirmations for self-compassion.

I actually had an eating disorder since I was 13 and when I was in my early 20s I went and checked myself into treatment for that and when I showed up there they had all these sticky notes on the doors and on the bathroom mirrors that had affirmations. With me being numb to emotions I was like this is the most stupid thing ever but overtime through practicing those affirmations and affirmations that my therapist gave me… I think that I might have been feeling compassion towards myself (Participant 15).

For this participant, practicing the affirmations they learned through their healthcare provider was a way towards self-compassion. When participants were able to show themselves compassion they said that it was life changing. Participant 16 noted that “it was self-compassion that helped me.” Self-compassion was also said to be important for “recharging” oneself against the hurts of the world. As Participant 14 described, “I think that self-compassion mirrors self-care… and I look at self-compassion as a way to recharge.” Through this lens, self-compassion was seen as a way to healing and health. These experiences indicated that meanings of self-compassion for many healthcare professionals are created through healthism discourses that place the responsibility for healing and improved health with the individual. It is up to 2SLGBTQ+ people to move past their open wounds, perform affirmations, and control their thinking in order to heal through self-compassion.

## Discussion

As the theologian Michael Fox (1999) suggested it is important for compassion to be analyzed and critiqued as a means to create knowledge surrounding how people can act in non-judgmental ways to help move through universal pain and suffering, as well as to create connections between people. We feel compassion research within health disciplines is therefore essential for more effective healthcare and patient wellbeing. The three discursive considerations provide insight into how cis-heteronormative and healthism discourses create healthcare systems that are often non-compassionate, particularly for 2SLGBTQ+ people. Fox (1999) critiqued the westernized medical system, noting that it has “… grown into a capital-intensive, quantitatively-orientated, ladder-orientated, violent business” that is lacking in compassion (p. 230). A study of mental health professionals showed that a “production-line mentality” was prevalent in the language they used and may reflect beliefs that productivity in care is valued more than compassion in care (Crawford et al., 2013). Productivity is central to healthism discourse. As previously stated, westernized health systems are at the core driven by healthism discourse and principles of individualism (McGregor, 2001) that often do not recognize societal challenges and trauma many 2SLGBTQ+ people face as a consequence of cis-heteronormative discourses. Health professionals who are not trained and not open to understanding 2SLGBTQ+ experiences are also a product of cis-heteronormative discourses and are barriers to improved health. How can health professionals expect 2SLGBTQ+ people to individually practice self-compassion if the healthcare system does not provide compassion to 2SLGBTQ+ people?

Yet our participants believed compassion was needed at the core of healthcare. Compassion was seen as feelings of comfort and safety that included careful use of language and the awareness and understanding of shared experiences of trauma. Fox (1999) noted that healing can be “the act of removing the obstacles to compassion” (p.xiv). Our participants, although not explicating using the language of Fox (1999), agreed that challenging and disrupting cis-heteronormative discourses, or removing the obstacles to compassion, would be a way to more effective healthcare for 2SLGBTQ+ people.

Assumptions of healthism also need to be challenged, disrupted, and subverted to allow compassion within healthcare systems. Self-compassion is recognized to be an integral component of compassion (Neff, 2003a). Many researchers reported the benefits of self-compassion to health (Dunne et al., 2018; Gedik, 2019; Phillips & Hine, 2021; Sirois et al., 2015; Terry & Leary, 2011) and our participants discussed how self-compassion helped them to deal with some of the traumas that they had experienced throughout their lives. Self-compassion seems to be a critical act of healing. However, self-compassion can also be viewed as an act of individual healthism. People are asked to perform acts of self-care to improve their health and wellbeing within a society in which unequal relations of power exist, and society is generally not compassionate to 2SLGBTQ+ people. Our participants discussed how hard it was to practice self-compassion when they had open wounds of trauma, were “othered,” and were the recipients of abuse, homophobia, and transphobia. As Leget (2015) argued “promoting compassion on an individual level can never be a solution for a healthcare system that fails to be humane” (p. 677). Social changes in relation to topics of sexuality, gender, and ethnicity need to occur for transformative changes in health. A few participants used the example of how the HIV/AIDS epidemic galvanized activists through their shared experiences of death and dying to address the apathy of political and health institutions. This activism was viewed, by the participants, as a way that society became more compassionate towards 2SLGBTQ+ people by learning and recognizing the realities of their lives.

We suggest that another change within healthism discourses that could be transformative is critiquing ingrained concepts such as internalized homophobia and internalized transphobia. These terms are used to explain how 2SLGBTQ+ people supposedly experience self-hatred and guilt about being 2SLGBTQ+ (Aguinaldo, 2008; Isacco et al., 2012). These terms have become common in both healthcare language and the popular understanding of the health of 2SLGBTQ+ people. The meanings of these terms, however, have significance and are problematic in the construction of an approach to compassionate 2SLGBTQ+ healthcare. As Aguinaldo (2008) clarifies, these terms represent an individualistic approach to 2SLGBTQ+ healthcare that does not fully examine relations of power nor the way social discourses create meanings and experiences for people. Internalized homophobia places the responsibility of health onto the 2SLGBTQ+ individual who is identified as the problem, who is othered and often who is viewed to have less power within healthcare systems (Aguinaldo, 2008; Isacco et al., 2012). Internalized homophobia positions 2SLGBTQ+ as psychologically damaged with a negative self-image. As a result, 2SLGBTQ+ people engage in coping strategies to offset the tensions produced by their inner damage (Aguinaldo, 2008). Coping strategies identified in the literature include drug and alcohol abuse, acts of violence, riskier sexual behaviors, poorer educational performance, and suicidality (Garofalo & Katz, 2001). As Aguinaldo (2008) further states, these concepts focus “our attention toward the dark workings of the mind” (p. 93) and not the social discourses that create a system that privileges and normalizes cis-heterosexuality. In other words, the language of internalized homophobia and internalized transphobia shifts the focus onto people, prompting healthcare interventions, such as affirmations and the fostering of self-compassion, to be aimed at the individual instead of changing the broader social structures that create detrimental health experiences of 2SLGBTQ+ people (Aguinaldo, 2008). The individualistic approach to the health of 2SLGBTQ+ people may not be truly compassionate care.

### Limitations

It should be noted that we have analyzed the data as a whole to recognize that all the groups within the 2SLGBTQ+ umbrella (in fact all people) are subjugated to cis-heteronormative discourses and healthism discourses. We recognize, however, that 2SLGBTQ+ groups are not all the same and that the degree to which these discourses shape the experiences of each group is different. For example, it has been well documented that sexual orientation, race, and ethnicity are intimately connected and cannot be separated in healthcare (Marshall et al., 2021). Therefore, individuals who hold multiple identities of marginalization face fundamentally different experiences than either white sexual minorities or heterosexual Black, Indigenous, and People of Color. Other researchers have shown that trans people are likely to experience a higher incidence of poverty, food insecurity, and barriers to employment, education, housing, and safe and affirming healthcare (Fergusson et al., 2018; Kcomt et al., 2020). Approximately, two-thirds of our participants self-identified as white and about 60% of our participants self-identified as cis. Therefore, there are many voices not captured in our data. Further research could explore the meanings and experiences of compassion through a more intersectional lens to explore issues relating to race, gender, and other identities.

## Conclusion

This study only provides a glimpse into the meanings of compassionate healthcare for 2SLGBTQ+ people. As such, we cannot make wide sweeping generalizations nor recommendations. As previously mentioned, people within the 2SLGBTQ+ umbrella are diverse and have many different experiences in relation to healthcare. However, we can conclude that compassion was viewed in many ways by the participants of this study. It encompassed principles of safety, awareness of language, and the recognition of shared trauma that many 2SLGBTQ+ experience as part of their lives. Compassion was viewed as a central and critical component for healthcare and health professionals to enact as part of optimal care. We suggest, through our poststructural theoretical lens, that in order to truly transform healthcare, professionals must explore, examine, critique, and challenge how social discourses of sexuality, gender, and individualism create and recreate healthcare practices, institutions, and systems. By doing this, all people, regardless of sexual or gender identity may find more compassion and better health and wellbeing.
